# Varying levels of X chromosome coalescence in female somatic cells alters the balance of X-linked dosage compensation and is implicated in female-dominant systemic lupus erythematosus

**DOI:** 10.1038/s41598-019-44229-9

**Published:** 2019-05-29

**Authors:** Agnieszka I. Laskowski, Daniel S. Neems, Kyle Laster, Chelsee Strojny-Okyere, Ellen L. Rice, Iwona M. Konieczna, Jessica H. Voss, James M. Mathew, Joseph R. Leventhal, Rosalind Ramsey-Goldman, Erica D. Smith, Steven T. Kosak

**Affiliations:** 1 Department of Cell and Molecular Biology, Feinberg School of Medicine, Northwestern University, Chicago, IL 60611 USA; 2 Comprehensive Transplant Center, Department of Medicine, Surgery Division, Feinberg School of Medicine, Northwestern University, Chicago, IL 60611 USA; 3 Deparment of Medicine, Rheumatology Division, Feinberg School of Medicine, Northwestern University, Chicago, IL 60611 USA

**Keywords:** Epigenetics, Chromatin

## Abstract

The three-dimensional organization of the genome in mammalian interphase nuclei is intrinsically linked to the regulation of gene expression. Whole chromosome territories and their encoded gene loci occupy preferential positions within the nucleus that changes according to the expression profile of a given cell lineage or stage. To further illuminate the relationship between chromosome organization, epigenetic environment, and gene expression, here we examine the functional organization of chromosome X and corresponding X-linked genes in a variety of healthy human and disease state X diploid (XX) cells. We observe high frequencies of homologous chromosome X colocalization (or coalescence), typically associated with initiation of X-chromosome inactivation, occurring in XX cells outside of early embryogenesis. Moreover, during chromosome X coalescence significant changes in Xist, H3K27me3, and X-linked gene expression occur, suggesting the potential exchange of gene regulatory information between the active and inactive X chromosomes. We also observe significant differences in chromosome X coalescence in disease-implicated lymphocytes isolated from systemic lupus erythematosus (SLE) patients compared to healthy controls. These results demonstrate that X chromosomes can functionally interact outside of embryogenesis when X inactivation is initiated and suggest a potential gene regulatory mechanism aberration underlying the increased frequency of autoimmunity in XX individuals.

## Introduction

The three-dimensional (3D) organization of whole chromosomes and their encoded gene loci in the interphase nucleus is intrinsically linked to gene regulation. During interphase the 46 chromosomes of the human genome are partially de-condensed and organized into individual rough spherical shapes 2–4 µm in diameter, referred to as chromosome territories (CTs)^1^. CTs neighbor one another to form an interchromatin compartment, although boundaries between neighboring CTs are not rigid but rather intermingle at their peripheries^2,3^. Importantly, CTs occupy non-random spatial positions based upon gene density, size, and coordinate gene regulation, the latter of which results in lineage-specific genome topologies^1,4,5^. For example, gene-dense CTs and active genes are preferentially located within the nuclear interior while silenced chromatin and gene-poor CTs localize to the nuclear periphery. Dynamic changes to nuclear organization coincide with changes in cellular identity such as differentiation. During differentiation, co-mingling of CTs and particular gene loci to common transcription hubs facilitates rapid coordinate gene expression^4,6^. Moreover, homologous chromosomes also demonstrate a significant propensity to associate that facilitates coordinate gene expression during differentiation^7,8^, highlighting the importance of CT organization in the 3D nuclear space for gene regulation.

A natural occurring model for the effects of homologous chromosome association is X-chromosome inactivation (XCI). To establish equivalent X-linked gene expression between human male (46, XY) and female (46, XX) cells, XCI is initiated during early embryogenesis in human female cells^9,10^. Either the maternal or paternal derived X chromosome is randomly selected for whole chromosome transcriptional silencing^11^. During XCI the two X chromosomes colocalize (or coalesce) in the nuclear periphery of the interphase nucleus, utilizing *trans*-regulatory mechanisms to ensure only one X chromosome is selected for silencing while the other remains active^12,13^. Xist, a 19 kb long non-coding (lnc) RNA, is expressed from the X chromosome selected for inactivation^14–16^. Xist spreads in *cis* to transcriptionally active regions, recruiting epigenetic and chromatin conformation silencing mechanisms^17,18^. Shortly after the Xist RNA accumulates on the inactive X chromosome (Xi), histone modifications associated with gene expression are lost^19–24^. Next, Xist recruits repressive protein complexes PRC2, HBiX1, and SMCHD1, resulting in repressive epigenetic modifications on histone H3, including K27me3 and K9me3^25–27^, as well as PRC1 recruitment for the H2AK119 ubiquitination repressive mark^28,29^. The Xi undergoes whole-chromosome condensation facilitated by heterochromatin protein HP1, forming the transcriptionally inactive Barr body^30^. The opposing gene regulatory environments of the active X chromosome (Xa) and Xi are maintained during all subsequent cell divisions^31^. However, approximately 5% of X-linked genes on the Xi escape XCI, and an additional 10% have variable patterns of Xi escape^32,33^. Healthy male nuclei do not initiate XCI or express Xist due to the presence of only one X chromosome, although male individuals with human polysomy X, such as Klinefelter syndrome (47, XXY), do undergo XCI^34–36^. X-chromosome inactivation is a clear example of *trans-* and *cis-*regulatory mechanisms working collectively to achieve transcriptional balance.

In addition to providing a model for the effects of chromosome interaction, the presence of two homologous X chromosomes with transcriptionally converse epigenetic environments—Xa and Xi—is linked to an increased predisposition and progression for autoimmune disease. Among the multiple examples of female-dominant health disorders, this correlation may not be surprising as the X chromosome contains many immune-related genes^37^. Mouse genetic studies have demonstrated that the presence of X diploidy (XX) specifically exacerbates the autoimmune responses of multiple sclerosis (MS) and systemic lupus erythematosus (SLE), and this increased autoimmunity is not connected to the presence of female or absence of male gonads^38^. Thus, autoimmune diseases are more prevalent in women than in men, with SLE nine times more common in females^39,40^, due to the presence of two X chromosomes. This predisposition is further supported by the increased incidence of SLE in males with Klinefelter’s syndrome (47, XXY), in which SLE prevalence is equivalent to normal females (46, XX)^41^, as well as lowered risk of SLE in women with Turner syndrome (45, X)^42^.

Here we utilize the X diploidy of female cells as a model for testing the effects of chromosome interaction on gene regulation, with the Xa and Xi representing a naturally occurring epigenetic binary of active and inactive environments, respectively. Surprisingly, we identify high degrees of chromosome X coalescence in a variety of healthy human female (46, XX) and male Klinefelter syndrome (47, XXY) somatic cell types, greater than the levels observed in the early stages of human embryogenesis during XCI. Due to the opposing epigenetic and transcriptional characteristics of Xa versus Xi, we investigated the epigenetic consequences of X chromosome coalescence in somatic cells and determined significant shifts in Xist and H3K27me3 coverage of coalesced X chromosomes. Importantly, whole chromosome X-linked gene expression is also altered during X coalescence. These results suggest potential exchange of epigenetic and gene regulatory information between the Xa and Xi throughout the life span of XX cells. Due to the high frequency of chromosome X coalescence we observed in XX T lymphocytes and the known predisposition of SLE in women, we examined the chromosome X organization patterns of SLE T lymphocytes. We observed a significant disruption in chromosome X organization in SLE patient lymphocytes. Specifically, subsets of CD4+ T lymphocytes known to play an important role in SLE disease state progression have significantly lowered levels of X chromosome coalescence. Together, our results highlight a gene regulatory relationship between Xa and Xi chromosomes occurring outside of early human embryogenesis, and they indicate a disruption of chromosome X organization may be related to the loss of proper X-linked gene expression predisposing females (46, XX) and Klinefelter syndrome (47, XXY) individuals to autoimmunity.

## Results

### Homologous X chromosomes coalesce outside of XCI initiation in human XX cells

**Figure 1:**
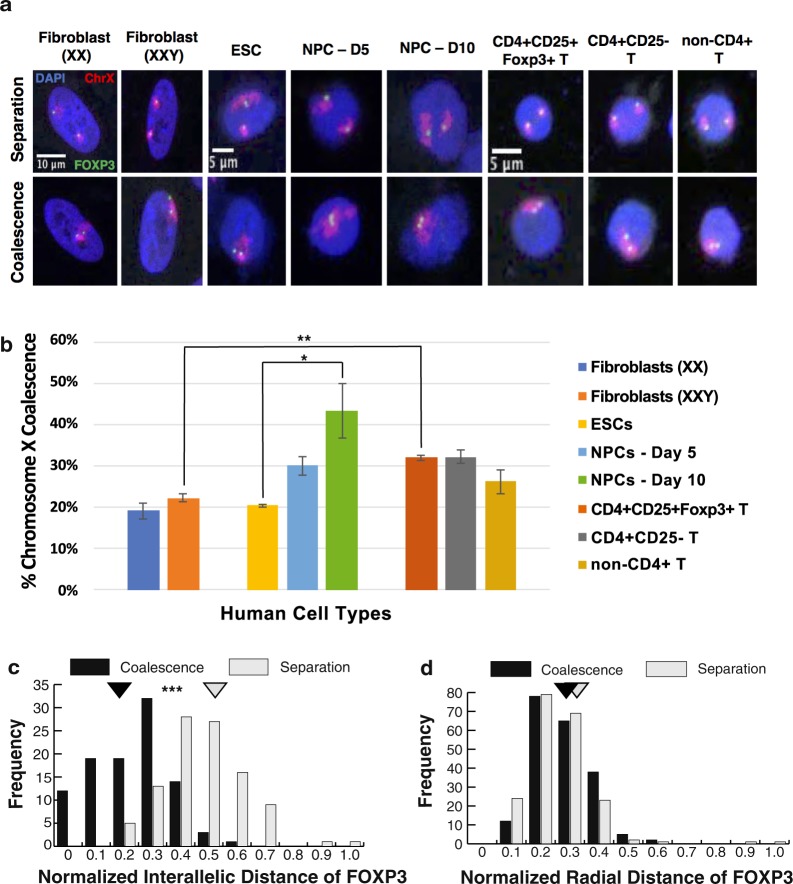
Chromosome X coalescence occurs at various frequencies in human cell types. (**a**) 3D DNA FISH maximum intensity projections of human nuclei labeled with DAPI (blue), chromosome X (red), and X-linked gene  locus, FOXP3 (green) in various human cell types containing two X chromosomes, during X separation (upper panel) or X coalescence (lower panel). (**b**) 3D analysis of chromosome X coalescence frequency in various human cell types. Values presented as mean ± standard deviation. *P < 0.05, **P < 0.01, Student’s *t*-test. Each column represents approximately 200–400 nuclei analyzed. Distribution display of normalized interallelic distances of FOXP3 (**c**) and normalized radial distances of FOXP3 (**d**) during either X coalescence or separation in human regulatory T cells (CD4+ CD25+ Foxp3+ T). Normalized distance = [interallelic: FOXP3 loci to FOXP3 loci] or [radial: FOXP3 loci to nuclear periphery] distance/d, where d = 2 × (nuclear area/π)^0.5^. Normalized distance ranges from 0 to 1. Mean normalized distances, triangles. *P < 0.05, **P < 0.01, ***P < 0.001, Student’s *t*-test. A total of 200 FOXP3 loci were analyzed during X coalescence and X separation for interallelic and radial distances. ESC(s): embryonic stem cell(s), NPC(s): neural precursor cells.

To assay chromosome X nuclear organization outside of early embryogenesis and XCI initiation, we first visualized whole chromosome X territories in human female (IMR-90, 46, XX) and phenotypically male fibroblasts (Klinefelter syndrome, 47, XXY) during interphase with DNA fluorescence *in situ* hybridization (FISH) followed by three-dimensional (3D) microscopy. Surprisingly, ~20% of the nuclei from both lines of fibroblasts have coalesced X chromosomes (Fig. 1a,b). Thus, XX chromosome association occurs to an unexpected degree in differentiated cells and the presence of an additional sex chromosome (Y) does not affect their capacity to coalesce. As indicated above the only reported occurrence of X-chromosome pairing occurs during embryogenesis. For example, during mouse embryonic stem cell (mESC) differentiation toward a neuronal cell fate, X coalescence has been reported to occur during a 6-day timeline concurrent with initiation of XCI^12,13^. Therefore, we analyzed the differentiation of human embryonic stem cells (hESCs) (WA-09 cell line) toward the same neuronal progenitor cell (NPC) fate and unexpectedly identified high levels of X coalescence up to 10-days post induction (Fig. 1a,b). Moreover, chromosome X coalescence gradually and significantly increased over the differentiation time course with a pronounced degree of association in NPCs (Fig. 1a,b). These results demonstrate that the association of X chromosomes can occur outside of embryogenesis with levels of coalescence varying among cell lineages.

Due to their role in autoimmune development and progression, we furthered our analysis of X-chromosome organization in human female T lymphocytes: including, regulatory T cells (Tregs) (CD4+ CD25+ Foxp3+), CD4+ CD25− T, and non-CD4+ T lymphocytes. Tregs play an important role in suppressing autoimmunity^43–45^ and this function is dependent on proper expression of FOXP3, a critical transcriptional regulator of Tregs, which is located on the X chromosome (Fig. 1a,b). Approximately 30% of all healthy human female T lymphocytes have coalesced X chromosomes (Fig. 1a,b), levels significantly greater than in female and Klinefelter syndrome fibroblasts. In addition, chromosome X coalescence occurs in mouse T lymphocytes and keratinocytes, highlighting conservation of homologous chromosome X nuclear organization during evolution (Supplemental Fig. 1a,b). Importantly, analysis of homologous coalescence of an autosome (chromosome 2) does not reveal a significant difference among the various cell types with the most pronounced and varied X coalescence levels (Supplemental Fig. 2a,b). Thus, X chromosome coalescence levels are not simply a reflection of the genome organization of these lineages, but specific to the organization of the X itself. Together these results point to varying levels of homologous chromosome X association in (XX) cells outside of early embryogenesis and XCI, and they suggest a potential regulatory role for X pairing in somatic cell types.

To determine the effects of X coalescence on nuclear organization traits that are linked to gene expression, we measured interallelic distance (distance between alleles on homologous chromosomes) and radial distance (allele distance to nuclear periphery) for the *FOXP3* genes in human female Tregs (Fig. 1c,d). During chromosome X coalescence, *FOXP3* alleles located on either the Xa or Xi chromosome are on average within 1.57 microns of one another. In Osborne *et al*.^6^, genes within 0.5 microns occupied shared transcriptional hubs. In our analysis of X coalescence in human Tregs, 12% of *FOXP3* homologous gene loci associated with converse epigenetic environments are within 0.5 microns of one another, well within the range of a shared transcriptional environment. Intriguingly, the radial distance of the *FOXP3* gene loci are unaffected by chromosome X coalescence, indicating a restricted radial distance positioning of *FOXP3* within the nucleus (Fig. 1d). These results demonstrate a unique chromosome X and X-linked gene locus organization with gene regulatory implications occurring in human female (46, XX) and male (47, XXY) cells that do not occur in normal male (46, XY) and Turner syndrome (45, X) cells.

### Xist coverage of X chromosomes is altered during coalescence

**Figure 2:**
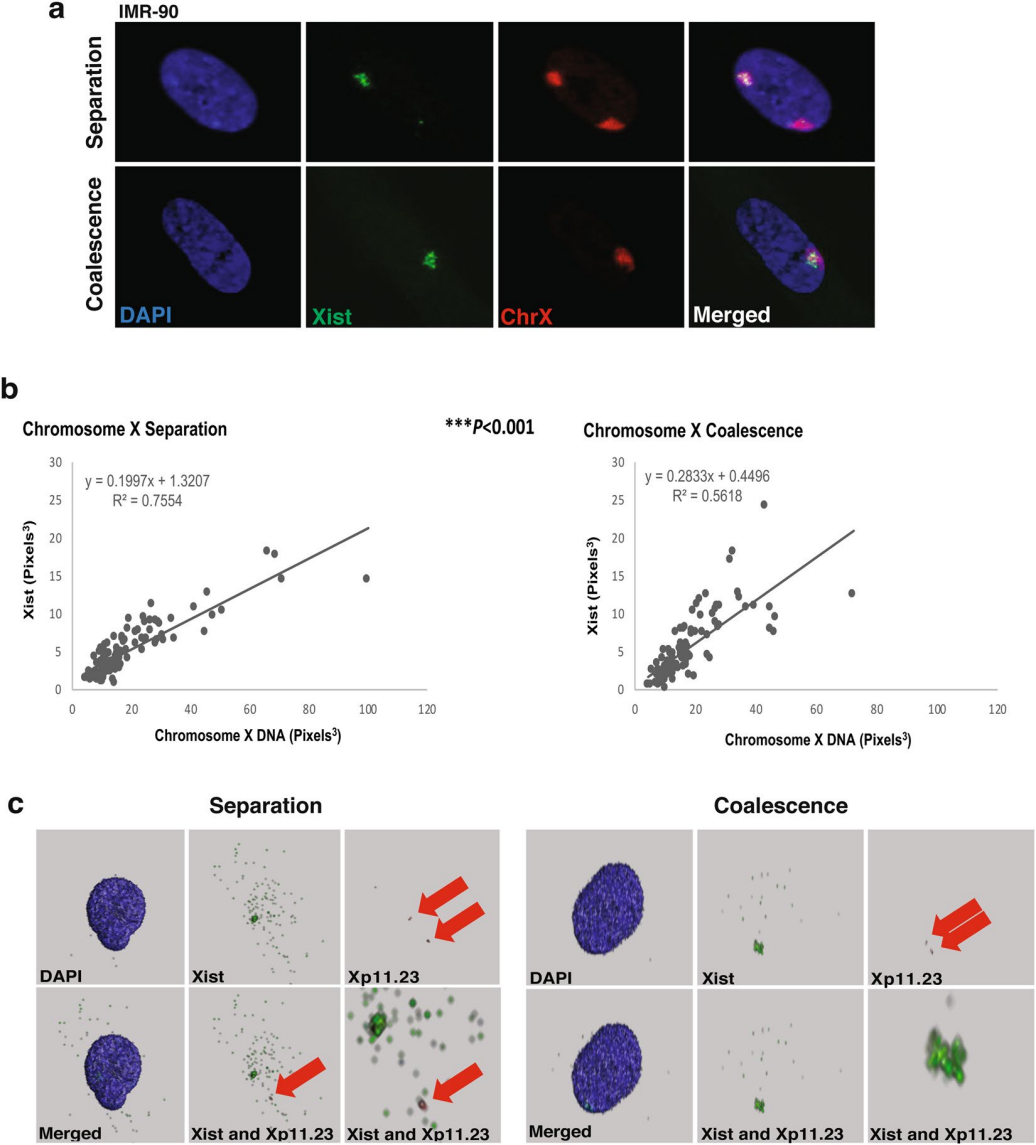
Xist coverage of chromosome X changes, during X coalescence in human female cells. (**a**) 3D maximum intensity projections of human IMR-90 nuclei (blue) with Xist (green) and chromosome X (red) labeled during X separation (upper panel) and coalescence (lower panel). (**b**) 3D volumetric measurements of Xist and total chromosome X, each data point represents measurements from one nucleus. A total of 100 nuclei analyzed during X separation and coalescence. *P < 0.05, **P < 0.01, ***P < 0.001, linear regression *t*-test. (**c**) 3D renderings of IMR-90 nuclei (blue) with Xist (green) and Xp11.23 loci (red) labeled during chromosome X separation (left panels) and chromosome X coalescence (right panels). Red arrows highlight visible Xp11.23 loci.

Due to the close proximity of the active gene regulatory environment of Xa and the repressive environment of Xi, exposure of X-linked genes to opposing regulatory mechanisms may occur during X coalescence. With the ability to delineate the location of repressive gene regulatory factors of Xi by localization of Xist, we simultaneously visualized Xist and chromosome X by 3D interphase RNA/DNA FISH in human female (46, XX) fibroblasts (Fig. 2a). Coverage of Xist over total chromosome X territories was measured in 3D during either X coalescence or separation. This analysis revealed Xist coverage of chromosome X is significantly altered when X chromosomes are coalesced versus separated (Fig. 2b). To ensure significant conformational changes to coalesced X chromosomes is not responsible for the altered relationship of Xist and chromosome X during coalescence, nuclear volume occupation of total chromosome X was measured in 3D during coalescence and separation. Total chromosome X volume occupation in the 3D nucleus is equivalent during separation and coalescence (Supplemental Fig. 3a). In addition, no significant difference between the volume of Xa and Xi is detected during X separation (Supplemental Fig. 3b) as previously described by Eils *et al*.^46^. Thus, changes in Xist coverage is due to localization changes of Xist on coalesced X chromosomes as opposed to changes in chromosome X conformation. Moreover, instances of Xist colocalization to the Xa are observed when homologous gene loci *FOXP3* (Xp11.23) overlap with Xist during coalescence in 3D (Fig. 2c). Our results demonstrate that Xist is capable of colocalizing over both the active and inactive X chromosomes during coalescence and raises the possibility of *trans*-chromosomal regulation between homologous X chromosomes.

**Figure 3:**
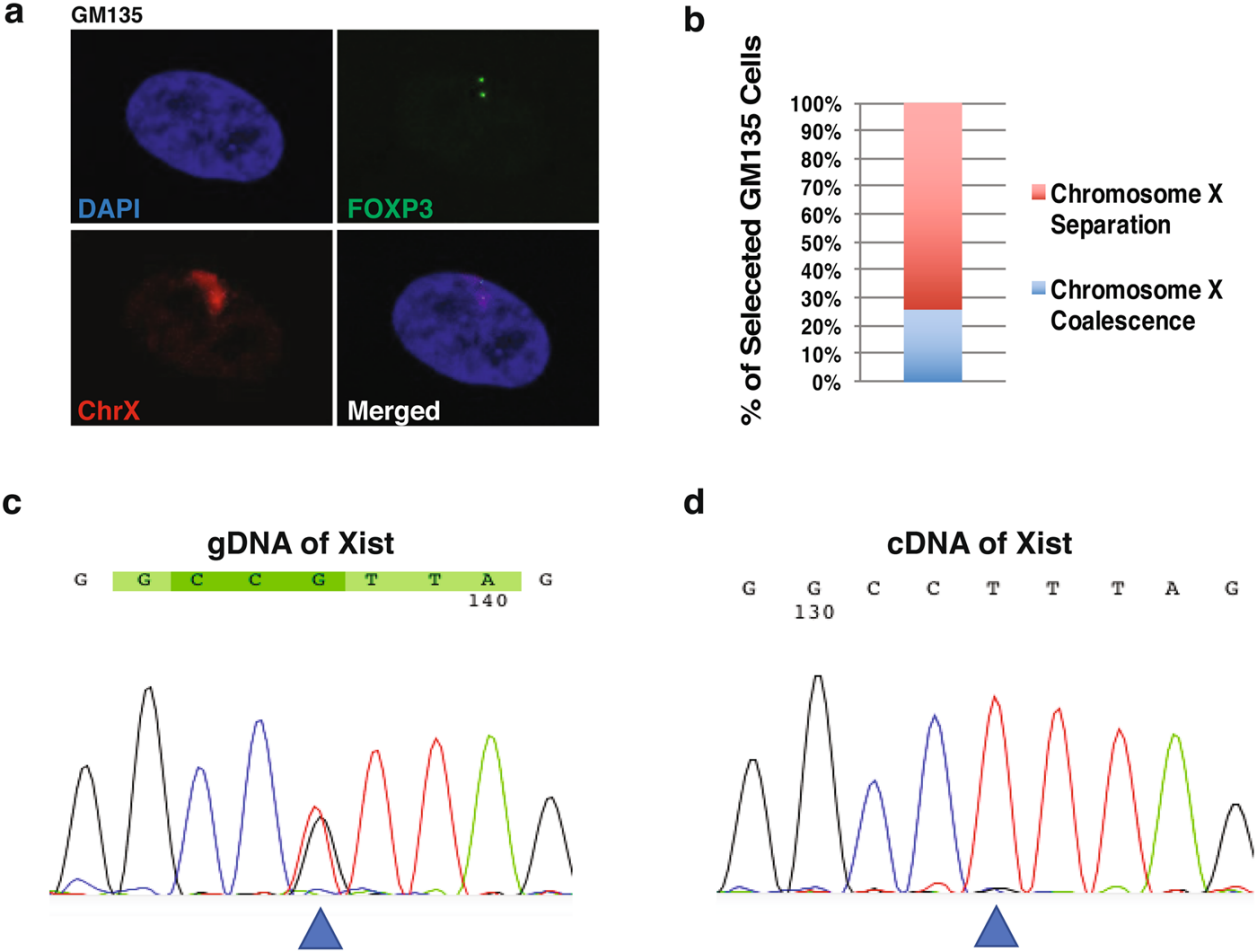
During X coalescence, Xist localization changes on the X chromosomes as opposed to Xist gene loci reactivation on the active X chromosome. (**a**) 3D DNA FISH maximum intensity projections of an 8-azaguanine selected human GM135 nuclei labeled with DAPI (blue), chromosome X (red), and X-linked gene loci, FOXP3 (green) during X coalescence. (**b**) Frequency of chromosome X coalescence in selected GM135 cells, 100 nuclei analyzed. (**c**) Xist genomic sequence containing a known single nucleotide polymorphism site (SNP rs16992443) (blue triangle) isolated from selected GM135 cells. (**d**) cDNA sequence of Xist, generated from RNA, isolated from selected GM135 cells. Location of known Xist SNP site (blue triangle).

Although we suggest the significant changes in Xist coverage during coalescence is due to localization changes of Xist and X-linked gene loci, it is also possible that the *XIST* allele on the Xa is reactivated. To determine whether Xist can be reactivated during X coalescence, we used the GM135 fibroblast cell line isolated from a female heterozygous for the mutated HPRT allele associated with Lesch-Nyhan syndrome. All human female cell lines are typically a heterogeneous mixture of both maternal and paternal derived X chromosomes being inactivated. The GM135 cell line, however, can be selected for a pure population of cells expressing the maternally derived X chromosome after incubation with 8-azaguanine^47,48^. GM135 cells also have a single nucleotide polymorphism (SNP) present in the XIST gene locus (Fig. 3c)^48^, allowing us to determine which XIST allele is being expressed. Therefore, we first selected GM135 cells and identified the presence of X coalescence by 3D DNA FISH (Fig. 3a,b). Post-selection, approximately 26% of GM135 cells have coalesced X chromosomes (Fig. 3b), yet only one version of the XIST SNP is expressed (Fig. 3d). During X coalescence, localization changes of Xist and X-linked gene loci in the 3D nuclear environment are responsible for significant alterations in Xist and chromosome X coverage as opposed to reactivation of the silenced *XIST* locus on the Xa. If reactivation of the *XIST* locus occurred, both *XIST* SNPs would be detected in cDNA generated from selected GM135 cells (Fig. 3d).

### Repressive epigenetic mark H3K27me3 is significantly altered upon X coalescence

**Figure 4:**
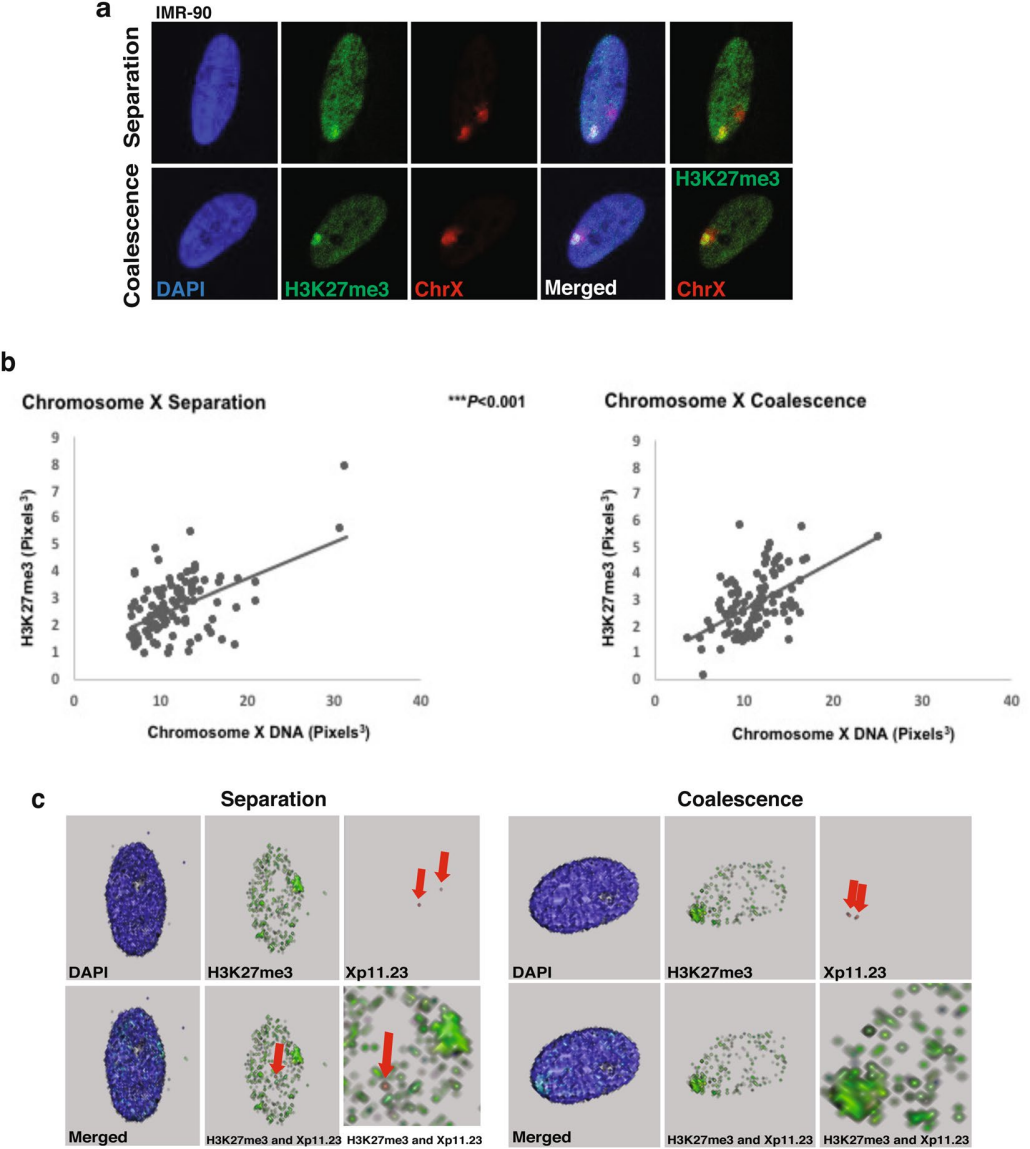
H3K27me3 coverage of chromosome X changes, during X coalescence in human female cells. (**a**) 3D maximum intensity projections of human IMR-90 nuclei (blue) with H3K27me3 (green) and chromosome X (red) labeled during X separation (upper panel) and coalescence (lower panel). (**b**) 3D volumetric measurements of concentrated H3K27me3 and total chromosome X, each data point represents one nucleus. A total of 100 nuclei analyzed during X separation and coalescence. *P < 0.05, **P < 0.01, ***P < 0.001, Welch two sample *t*-test. (**c**) 3D renderings of IMR-90 nuclei (blue) with H3K27me3 (green) and Xp11.23 loci (red) labeled during chromosome X separation (left panel) and chromosome X coalescence (right panel). Red arrows highlight visible Xp11.23 loci. Bottom right images are magnifications of bottom center images.

To determine whether changes in Xist coverage also alters the epigenetic landscape of coalesced X chromosomes, 3D interphase immuno-DNA FISH was carried out for H3K27me3 and chromosome X in human female (46, XX) fibroblasts (Fig. 4a). During XCI and maintenance Xist recruits a repressive protein complex (PRC2), resulting in repressive H3K27me3 epigenetic modifications^25,26^. H3K27me3 coverage of chromosome X is significantly different between X chromosomes that are separate versus coalesced (Fig. 4b). As observed with Xist, evidence of homologous gene loci of position Xp11.23 colocalize with H3K27me3 localization during X coalescence in 3D (Figs 2c and 4c). These results exhibit replacement of the Xa-linked genes to hubs of non-transcribed chromatin and further support the presence of potential epigenetic based *trans*-chromosomal gene regulation occurring during X coalescence in human female cells.

### Total chromosome X RNA expression is reduced by X coalescence

**Figure 5:**
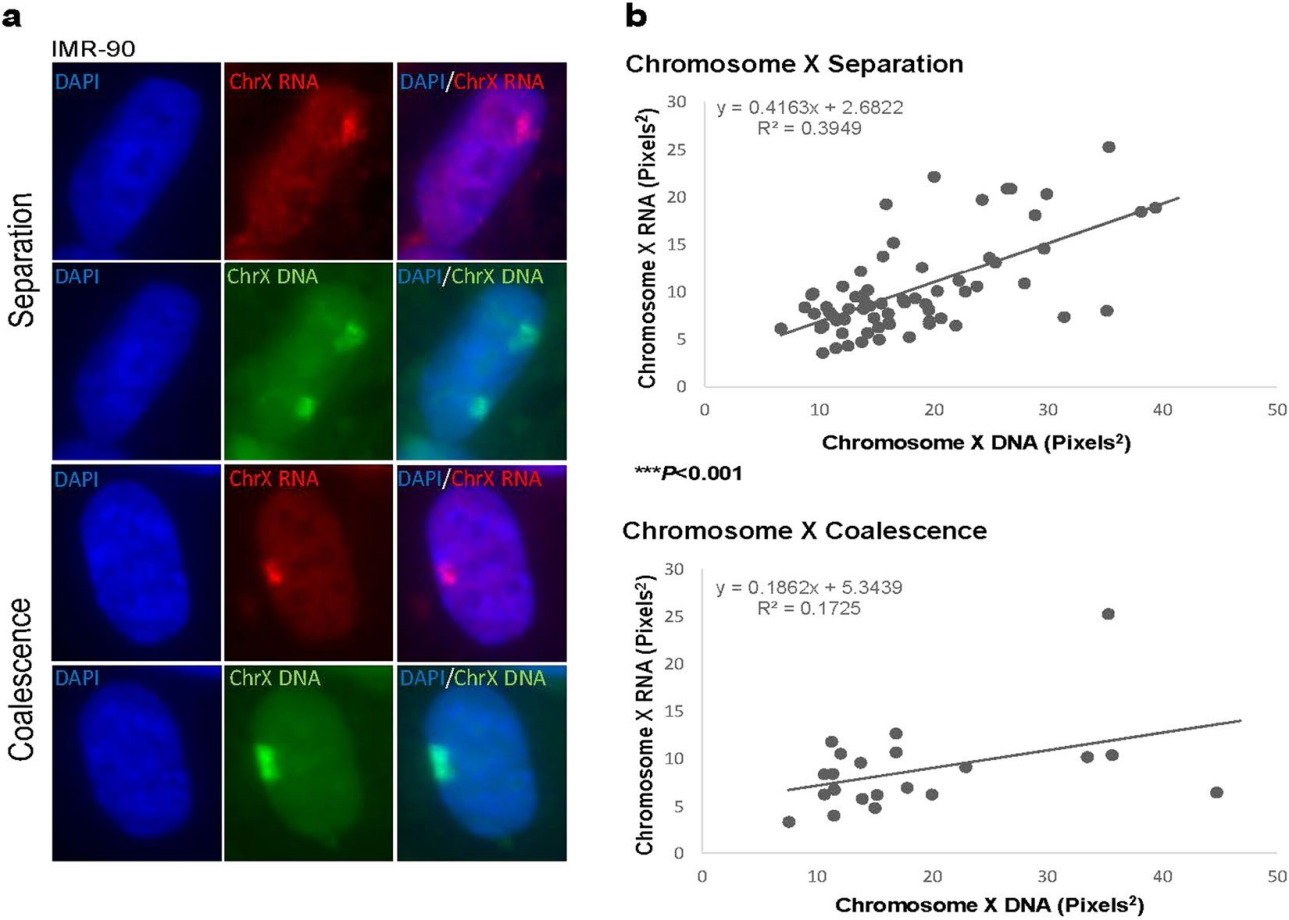
Total chromosome X expression is reduced during X coalescence. (**a**) Single z-section of human IMR-90 nuclei (blue) with chromosome X RNA (red) and chromosome X DNA (green) labeled during X separation (upper two panels) and coalescence (lower two panels). (**b**) Maximum intensity projection area measurements of total chromosome X RNA and total chromosome X DNA, each data point represents measurements from one nucleus. A total of 70 nuclei during X separation and 30 nuclei during X coalescence were analyzed. *P < 0.05, **P < 0.01, ***P < 0.001, Welch 2 sample *t*-test.

To determine the effects of chromosome X coalescence on X-linked gene expression, sequential RNA/DNA FISH was carried out for whole chromosome X RNA and DNA in human female (46, XX) fibroblasts (Fig. 5a). We followed the protocol optimized by *Vallot et al*.^49,50^ to identify changes in whole chromosome X expression on a per nucleus basis in XX pluripotent stem cells with minimal modifications. As observed with Xist and H3K27me3, whole chromosome X gene expression significantly changes when X chromosomes are coalesced (Figs 2b, 4b and 5b), resulting in significant reductions in whole chromosome X RNA detection (Fig. 5b). These results conform to our supposition of a gene regulatory mechanism occurring during X coalescence in X diploid cells resulting in modulation of X-linked gene expression.

### SLE CD4+ T lymphocytes have lowered levels of X coalescence

**Figure 6:**
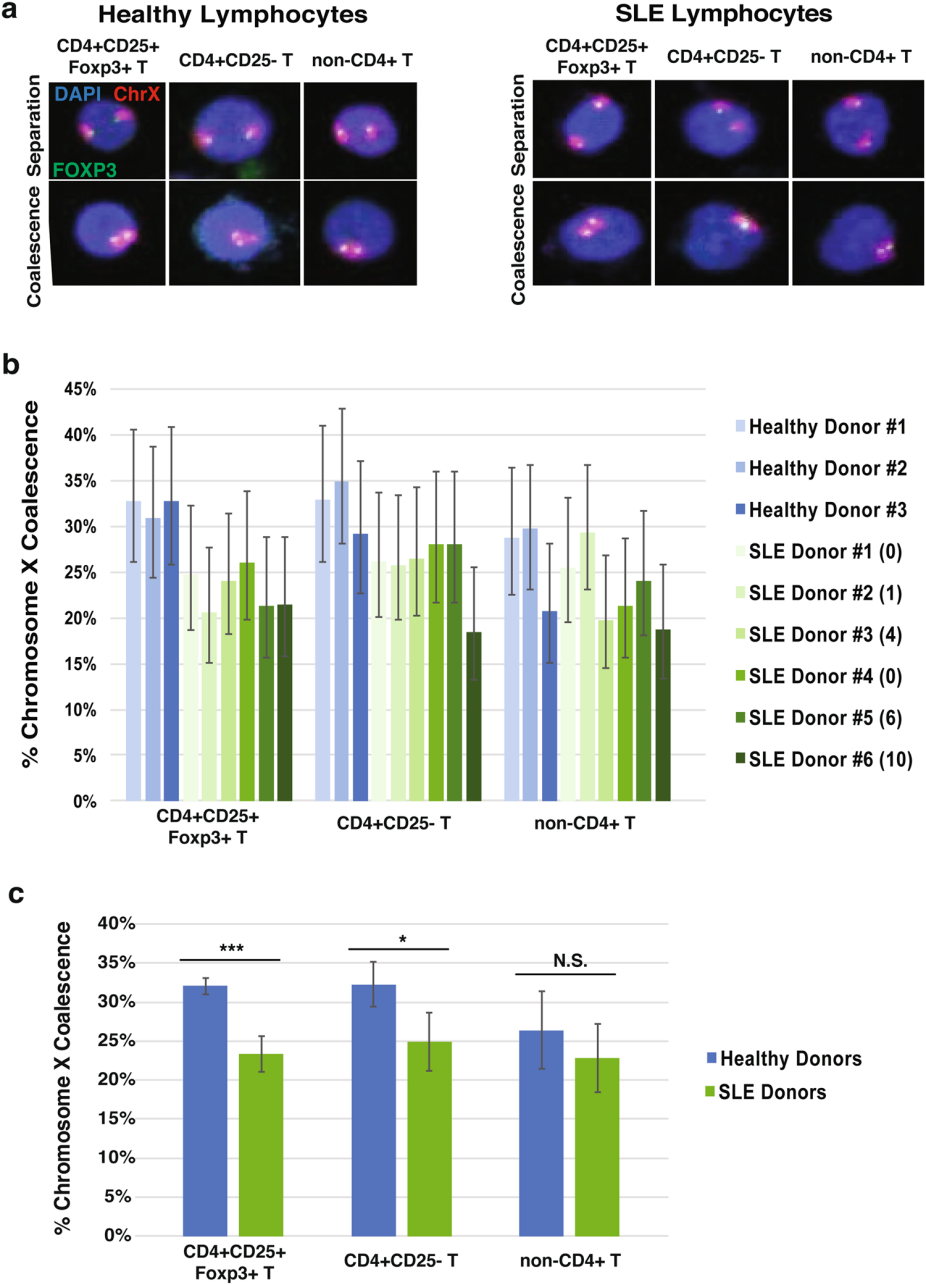
Human female systemic lupus erythematosus (SLE) CD4+ T lymphocytes exhibit significant differences in chromosome X organization compared to healthy controls. (**a**) 3D maximum intensity projections of human female healthy (left panels) and SLE (right panels) lymphocyte nuclei (blue) with FOXP3 (green) and chromosome X (red) labeled during X separation (top panels) and coalescence (bottom panels). (**b**) 3D chromosome X coalescence frequency of healthy and SLE regulatory T cells (CD4 + CD25 + Foxp3+), CD4 + CD25- T cells, and non-CD4+ T cells. Each column represents approximately 150 nuclei analyzed, error bars designate 95% confidence intervals. SLEDAI-2 donor score given in parenthesis. (**c**) Chromosome X coalescence frequency of healthy and SLE regulatory T cells (CD4 + CD25 + Foxp3+), CD4 + CD25- T cells, and non-CD4+ T cells. Values presented as mean ± standard deviation. *P < 0.05, **P < 0.01, ***P < 0.001, NS. = Not Significant, Student’s *t*-test. Each column represents approximately 450 healthy or 900 SLE nuclei analyzed.

Systemic lupus erythematosus (SLE) is nine times more common in women than in men, indicating an extreme sex predisposition toward XX individuals. A central mechanism of autoimmune disease involves the inappropriate activation of the adaptive immune system against self-antigens, leading to inflammation and damage to various tissues of the body. SLE involves inappropriate induction of CD4+ helper T cells and CD8+ effector T cells, followed by secretion of pro-inflammatory cytokines and chemokines^51^. A subset of T cells, regulatory T cells (Tregs), contribute to SLE by loss of their suppressive function of effector T cells^43–45^. To determine the chromosome X organization patterns in the context of autoimmune disease, we isolated T lymphocytes from human female inactive and active SLE donors. SLE disease is not a static illness but rather oscillates between active and inactive states. Inactive SLE disease is defined as a SLEDAI-2 score less than 6, and active SLE as greater than or equal to 6^52,53^. SLEDAI-2 scores are determined by severity of physiological symptoms, signs, and laboratory abnormalities caused by the illness^53^. In total three different T lymphocyte populations were analyzed from healthy, inactive, and active SLE patients: Tregs (CD4+ CD25+ Foxp3+), CD4+ CD25− T, and CD4− lymphocytes with SLEDAI-2 donor scores denoted (Fig. 6a). CD4- lymphocytes consist of cells with the CD8, CD19, CD123, and CD127 surface markers. Our analysis reveals a significant decrease in X coalescence frequency between healthy and disease state T cells, specifically in the CD4+ T population that includes Tregs (Fig. 6b,c). While it is clear that the presence of illness impacts X coalescence, the difficulty in acquiring patient donors means our sample size is small [(data points reflect ~2.5 years of patient recruitment) as many potential donor candidates had to be excluded due to treatment plans]. Nevertheless, SLE patients reveal a significant tendency for reduced X coalescence relative to healthy donors. To ensure medications were not responsible for differences in chromosome X coalescence, both inactive and active SLE donor medical treatments were examined and determined to have no overlap. These results support a unique organization of chromosome X homologues in the nuclei of autoimmune CD4+ T lymphocytes compared to healthy (non-lupus) controls. This phenomenon cannot occur in male diploid nuclei or Turner syndrome individuals (45, X) as only one X chromosome is present and chromosome X inactivation does not occur.

## Discussion

Our study reveals the unexpected finding that chromosome X coalescence occurs at significant levels in human XX cells outside of early embryogenesis and XCI (Fig. 1)^9,10,54^. Chromosome X coalescence levels vary by cell type, with fibroblasts, hESCs differentiated towards neural progenitors, and lymphocytes having different degrees of association (Fig. 1). These findings further differentiate the nuclear organization characteristics of normal human female and male cells. Due to XCI in XX cells, the Xi is a concentrated niche of transcriptional silencing mechanisms. During X coalescence the Xi and Xa gene loci are brought within close proximity of one another and thereby their converse transcriptional and epigenetic environments. Approximately 12% of *FOXP3* homologous gene loci and their opposing regulatory environments are within 0.5 microns of one another in human Tregs during X coalescence, well within the range of sharing a transcriptional environment. Importantly, proper expression of *FOXP3* is required for development and maintenance of Treg suppressive function. Mutation of the *FOXP3* gene in humans leads to immunodysregulation polyendocrinopathy enteropathy X-linked (IPEX) syndrome causing dysfunctional Tregs, severe autoimmunity, and premature death^55^. These studies highlight the importance of effective X-linked gene expression for proper immune mediated cellular function.

Xist and its tightly associated epigenetic mark, H3K27me3, exhibit significant alterations in chromosome X coverage during coalescence in human female cells (Figs 2 and 4). These results suggest localization of Xist and H3K27me3 incorporates into new areas of coalesced X chromosomes. Furthermore, they also indicate that coalescence brings both homologous gene loci Xp11.23 (*FOXP3*) into a chromatin environment enriched with H3K27me3 modifications (Figs 2c and 4c). Expression of one *XIST* SNP in selected human female carrier Lesch-Nyhan syndrome fibroblasts with coalescent X-chromosomes supports Xist localization changes occurring during coalescence as opposed to *XIST* reactivation on the Xa (Fig. 3). Moreover, significant disruptions in whole chromosome X gene expression further validates gene regulatory consequences occurring during X chromosome coalescence, suggesting *trans*-chromosomal gene repression of X chromosomes in human female somatic cell types (Fig. 5).

It is possible that X chromosomes come together throughout the life span of human female cells in order to exchange gene regulatory information between homologs. The strength of Xist silencing is manifest during targeted introduction of an *XIST* transgene. Xist alone is sufficient for silencing whole autosomes as seen in trisomy 21 induced pluripotent stem cells^56^. Also, an inducible human *XIST* cDNA is capable of silencing adjacent genes in somatic cells^57^, which is fully dependent on *XIST* expression since its loss reverses silencing^57^. In addition, mouse hematopoietic precursor cells have been found to be susceptible to Xist mediated gene silencing^58^. These studies support the principle that introduction of *XIST* expression is sufficient to induce gene repression of surrounding genes and its repressive nature is not limited to early development.

With 80% of all systemic autoimmune related disorders (SARDs) affecting women, the connection between X diploidy and propensity for autoimmunity has been postulated for some time^59^. In addition, X chromosomes contain the largest percentage of immune function genes in the entire genome and X-linked genes are known to be mutated or mis-regulated in SARDs patients^55,60–63^. However, the only potential mechanism between XX presence and autoimmunity has been identified as immune-related gene loci on the Xi escaping from X-inactivation in immortalized SLE B lymphocytes^64^. We aimed to determine whether chromosome X organization patterns in SARDs patient lymphocytes, specifically SLE T lymphocytes, may be different compared to healthy (non-lupus) controls. We have indeed identified significant differences in chromosome X coalescence levels in CD4+ T lymphocytes of inactive and active human female SLE patients (Fig. 6) compared to healthy (non-lupus) controls, particularly in Tregs (CD4+ CD25+ Foxp3+) and CD4+ CD25− T cell populations. Lowered levels of chromosome X contact in SLE CD4+ T lymphocytes may result in increased expression of immune-related genes on the X chromosome leading to dysfunctional immune cells and increased propensity for autoimmune disease development in XX cells. Identification of significant differences in chromosome X organization among human female healthy and SLE CD4+ T lymphocytes may provide a new and much-needed non-symptomatic diagnostic marker for SLE in women and men with Klinefelter syndrome. Moreover, efforts to identify the genes that promote or antagonize chromosome X pairing may provide novel drug targets for SLE treatment.

## Methods

### Cell culture

Primary human female lung fibroblasts (IMR-90, ATCC, Manassas, VA, USA), primary human male Klinefelter syndrome (XXY) fibroblasts (GM03102, Coriell Institute, Camden, NJ, USA), and primary human female carrier Lesh-Nyhan syndrome fibroblasts (GM00135, Coriell Institute) were cultured in DMEM alpha supplemented with 15% fetal bovine serum, 1% penicillin/streptomycin (100 U ml^−1^/100 μg ml^−1^) and 1% L-glutamine (2 mM) at 37 °C with 5% CO_2_. GM135 cells were selected with 8-azaguanine (1.5X stock) (MP Biomedicals, Santa Ana, CA, USA) in growth media, changed every 24 hr for 1 week.

The human embryonic stem cell (ESC) line WA-09 (WiCell, Madison, WI, USA) was used to generate committed neural precursor cells. The cell line was cultured feeder-free according to WiCell Research Institute recommended procedures using Matrigel and TeSR1 media at 37 °C with 5% CO_2_. Differentiated colonies were removed via aspiration after dispase treatment (2 mg/ml). Undifferentiated colonies were then scraped gently and grown in suspension media (1:1 DMEM/F12 + L-glutamine and Neurobasal media, 1x N2 and B27 supplements, 20 ng/ml insulin, 20 ng/ml bFGF, 20 ng/ml EGF) in non-tissue treated culture dishes for a period of 6 days, with intermittent 10 ml changes of suspension media every other day. Spheres were collected, gently triturated, and grown on polyornithine-coated (5 ng/ml for 1 hr at RT) tissue culture treated plates for an additional 4 days in expansion media (DMEM/F12 + L-glutamine, 10% BIT 9500, 20 ng/ml bFGF, 20 ng/ml EGF, 2ug/ml heparin), with 10 ml changes of media every other day.

According to the provisions set forth in the healthy control (IRB STU00002452) protocol and SLE patient (IRB STU00204004) protocol, donors informed consent was obtained prior to donation of 50–60 ml of peripheral blood (PB). Samples were given to the Immune Monitoring Laboratory (Northwestern University) for regulatory T cell (Treg, CD4^+^CD25^+^) isolation and characterization, CD4^+^CD25^−^ T and CD8^+^CD19^+^ T cells were also collected and analyzed. Peripheral blood mononuclear cells (PBMCs) were isolated from PB using lymphocyte separation media (Corning Inc, Corning, NY, USA) as previously described^65^. Using CD4+ CD25+ CD127dim regulatory T cell isolation kit and immunomagnetic isolation protocols (Miltenyi Biotec, Bergisch Gladbach, Germany), PBMCs were first depleted of CD8^+^ and CD19^+^ cells using an LS immunomagnetic column (Miltenyi Biotec), the total CD4+ fraction was collected. CD8^+^CD19^+^ cells were eluted from the column. Next, total CD4+ cell fractions were labeled with anti-CD25 PE beads (Miltenyi Biotec) to positively select for CD25^+^ expression (LS columns and autoMACs Running Buffer, Miltenyi Biotec). Cell fraction CD4^+^CD25^−^ passed through the immunomagnetic column and was collected whereas CD4^+^CD25^+^ Tregs were eluted from the column and collected for downstream analysis. After CD25 enrichment, 0.1 × 10^6^ Treg cells were stained with CD4 PeCy7 (BioLegend, San Diego, CA, USA), CD127 PE (BioLegend), and CD25 APC (Miltenyi Biotec) for 15 min at 4 °C. Cells were then fixed and stained for Foxp3 with anti-human mouse foxp3 fluorescein (Thermo Fisher Scientific) expression using the Foxp3/Transcription Factor Staining Buffer Set (Thermo Fisher Scientific). Samples were run on a Beckman Coulter 500 flow cytometer (Beckman Coulter, Brea, CA, USA) and data analyzed using FlowJo software (FlowJo, LLC, Ashland, OR, USA).

### Blood donor recruitment

All experimental procedures on human blood samples were approved by the Northwestern University Institutional Review Board (IRB) Committee. Healthy human female donors were recruited under Dr. Joseph Leventhal’s approved IRB protocol (STU00002452). Systemic lupus erythematosus (SLE) female donors were recruited under Dr. Rosalind Ramsey-Goldman’s approved IRB protocol (STU00204004). All guidelines and requirements outlined in the IRB protocols were followed. If donors agreed to participate and signed the patient informed consent form (approved by the IRB), about 50–60 ml of blood was obtained from the donors at Northwestern Memorial Hospital’s outpatient clinic. A trained technician introduced a sterile needle into a vein in the arm of the donor using sterile technique. For SLE donors, Dr. Ramsey-Goldman reviewed SLE patient medical records at the time of blood draw to determine the status of their disease activity and the types of medications they were currently taking. All patient donors were older than 18 years.

### Three-dimensional interphase DNA fluorescence *in situ* hybridization

DNA probes for FOXP3 were generated by nick translation (mix, Millipore Sigma, St. Louis, MO, USA) of the bacterial artificial chromosome (RP11-528A24, BACPAC Resource Center, Children’s Hospital Oakland Research Center, Oakland, CA, USA) and labeled with digoxigenin-11-dUTP (Millipore Sigma) according to the manufacturer. Human chromosome X paint is commercially available (XCP X Orange, MetaSystems, Heidelberg, Germany). Cells cultured in suspension were plated on sterile pre-coated poly-l-lysine (0.1%) (Millipore Sigma) coverslips prior to fixation. Adherent cells were incubated with sterile coverslips 24 hr prior to fixation. Standard 3D interphase DNA FISH protocol was performed based on the protocol of Cremer *et al*.^65^ of sections 3.2.1, 3.2.4, and 3.2.5. Minimal modifications to the DNA FISH protocol include 4 hr 20% glycerol incubation step, no liquid nitrogen freezing steps, 7 min 75 °C denaturation step, and FOXP3/chromosome X paint hybridization for 3 days.

### Three-dimensional interphase RNA/DNA fluorescence *in situ* hybridization

RNA probes for Xist were generated by nick translation (mix, Millipore Sigma) of an Xist plasmid (generously provided by A. Goldman and originally from E. Heard lab) and labeled with digoxigenin-11-dUTP (Millipore Sigma) according to the manufacturer. DNA probes for FOXP3 were generated by nick translation as previously described and labeled with DNP-11-dUTP (PerkinElmer, Waltham, MA, USA). Standard 3D interphase DNA FISH protocol was performed based on the protocol of Cremer *et al*.^66^ of sections 3.2.1, 3.2.4, and 3.2.5 with modifications to preserve RNA signal. All procedures were carried out under sterile/RNAse free conditions. All solutions were autoclaved and treated with diethyl pyrocarbonate (DEPC, Millipore Sigma). Hybridization solutions were supplemented with 1% VRC (200 mM ribonucleoside vanadyl complex, New England BioLabs, Ipswich, MA, USA) and block solutions supplemented with 10% VRC. Additional modifications include 4 hr 20% glycerol incubation step, no liquid nitrogen freezing steps, 7 min 75 °C denaturation step, and chromosome X paint with or without FOXP3 probes were hybridized overnight.

### RNA isolation, cDNA, PCR, and sequencing

Selected GM135 cells were harvested, washed, and pelleted, followed by total genomic DNA isolation with DNeasy blood and tissue kit (Qiagen, Hilden, Germany) or RNA isolation with TRIzol (Thermo Fisher Scientific, Waltham, MA, USA). cDNA was synthesized from RNA using the SuperScript^TM^ IV First-Strand Synthesis System (Thermo Fisher Scientific). The Xist gene region containing the single nucleotide polymorphism site (rs16992443) was amplified with the following primer pair: forward primer 5′ GTG ACA CAA GGC CAA CGA CC 3′ and reverse primer 5′ GAA TCA GGC TTA AAG ATA AAC AGG AG 3′ by PCR. PCR results were sequenced by AGCT, INC. (Wheeling, IL, USA).

### Three-dimensional interphase immuno-DNA fluorescence *in situ* hybridization

DNA probes for FOXP3 were generated by nick translation as previously described, in the three-dimensional interphase DNA fluorescence *in situ* hybridization section, however, probes were labeled with DNP-11-dUTP (PerkinElmer, Waltham, MA, USA). Standard 3D interphase immuno-DNA FISH protocol was performed based on the protocol of Cremer *et al*.^66^ of sections 3.2.6, 3.2.4, and 3.2.5. Minimal modifications include 2 hr 20% glycerol incubation step, no liquid nitrogen freezing steps, and chromosome X paint with or without FOXP3 probes were hybridized overnight.

### Whole chromosome X RNA/DNA fluorescence *in situ* hybridization

Successive RNA/DNA FISH with chromosome X paint (XCP X Orange; XCP X Green, MetaSystems) was performed based on the protocol of Vallot *et al*.^49,50^ with minimal modifications. A total of 6 µl of human chromosome X paint (XCP X Orange, MetaSystems) was co-precipitated with 1ug/µl Cot-1 DNA prior to RNA hybridization. Fibroblasts were plated on 22 × 22 mm square coverslips. Sequential imaging was performed on a Leica inverted Ti microscope (Leica, Wetzlar, Germany) with a motorized stage for high content imaging (optogenetic platform and camera; MetaMorph© Microscopy Automation). Images were analyzed with FIJI image analysis software (fiji.sc).

### Antibodies/Reagents

For DNA FISH, secondary antibody anti-sheep digoxigenin conjugated with fluorescein (Jackson ImmunoResearch Laboratories, West Grove, PA, USA) was diluted 1:250. For RNA/DNA FISH with FOXP3-DNP probes, rabbit anti-DNP antibody (Thermo Fisher Scientific) was diluted 1:1000. Next, secondary antibody sheep anti-digoxigenin conjugated with fluorescein (Jackson ImmunoResearch Laboratories) was diluted 1:250 and goat anti-rabbit alexa fluor 594 (Thermo Fisher Scientific) was diluted 1:1000. For immuno-DNA FISH, rabbit polyclonal antibody against H3K27me3 (Millipore Sigma) was diluted 1:50 followed by biotin conjugated anti-rabbit antibody (Thermo Fisher Scientific) was diluted 1:250 and secondary antibody anti-mouse biotin conjugated with fluorescein (Jackson ImmunoResearch Laboratories, West Grove, PA, USA) was diluted 1:250. For immuno-DNA FISH containing FOXP3-DNP probes, rabbit anti-DNP antibody (Thermo Fisher Scientific) was diluted 1:1000 and secondary goat anti-rabbit alexa fluor 594 (Thermo Fisher Scientific) was diluted 1:1000. A total of 8 µl of human chromosome X paint (XCP X Orange, MetaSystems) was hybridized per 22 × 22 mm square coverslip for fibroblasts and 6 µl was hybridized for the differentiation assay and lymphocytes plated on 12 mm round coverslips.

### Image acquisition and analysis

Slides were imaged on an A1R Resonant Scanning Multispectral Confocal microscope (Nikon, Tokyo, Japan) at the Northwestern University Center for Advanced Microscopy. Slides were imaged to encompass all nuclear signal in 0.2 µm z-stacks. Three-dimensional renderings of acquired images were analyzed with Image-Pro Plus software (Media Cybernetics, Rockville, MD, USA). Student’s *t*-test was performed for chromosome X coalescence frequencies; either liner regression *t*-test or Welch-2 sample *t*-test was performed for 3D image analysis with Welch-2 sample *t*-test performed in instances of unequal sample sizes.

## Supplementary information

Supplemental Info
