# Author Correction: Varying levels of X chromosome coalescence in female somatic cells alters the balance of X-linked dosage compensation and is implicated in female-dominant systemic lupus erythematosus

**DOI:** 10.1038/s41598-019-51828-z

**Published:** 2019-11-01

**Authors:** Agnieszka I. Laskowski, Daniel S. Neems, Kyle Laster, Chelsee Strojny-Okyere, Ellen L. Rice, Iwona M. Konieczna, Jessica H. Voss, James M. Mathew, Joseph R. Leventhal, Rosalind Ramsey-Goldman, Erica D. Smith, Steven T. Kosak

**Affiliations:** 1 Department of Cell and Molecular Biology, Feinberg School of Medicine, Northwestern University, Chicago, IL 60611 USA; 2 Comprehensive Transplant Center, Department of Medicine, Surgery Division, Feinberg School of Medicine, Northwestern University, Chicago, IL 60611 USA; 3 Deparment of Medicine, Rheumatology Division, Feinberg School of Medicine, Northwestern University, Chicago, IL 60611 USA

Correction to: *Scientific Reports* 10.1038/s41598-019-44229-9, published online 29 May 2019

This Article contains a repeated typographical error where

“SLEDAI-2”

should read:

“SLEDAI-2K”

