# Supplementary material for: Varying levels of X chromosome coalescence in female somatic cells alters the balance of X-linked dosage compensation and is implicated in female-dominant systemic lupus erythematosus
Source: Sci Rep. 2019 May 29;9:8011. doi: 10.1038/s41598-019-44229-9 (PMC6541617; doi:10.1038/s41598-019-44229-9)
Supplement: Supplementary file 1 — Supplemental Info [file 41598_2019_44229_MOESM1_ESM.pdf]

Varying levels of X chromosome coalescence in female somatic cells alters the balance of X-linked dosage compensation and is implicated in female-dominant systemic lupus erythematosus

Agnieszka I. Laskowski[1], Daniel S. Neems[1], Kyle Laster[1], Chelsee Strojny-Okyere[1], Ellen L. Rice[1], Iwona M. Konieczna[2], Jessica H. Voss[2], James M. Mathew[2], Joseph R. Leventhal[2], Rosalind Ramsey-Goldman[3], Erica D. Smith[1], and Steven T. Kosak[1]*

[1]Department of Cell and Molecular Biology, [2]Comprehensive Transplant Center, Department of Medicine, Surgery Division, [3]Deparment of Medicine, Rheumatology Division, Feinberg School of Medicine, Northwestern University, Chicago, IL 60611

*corresponding author, s-kosak@northwestern.edu, 312-503-9582

Supplemental Figures

Supplemental Figure 1

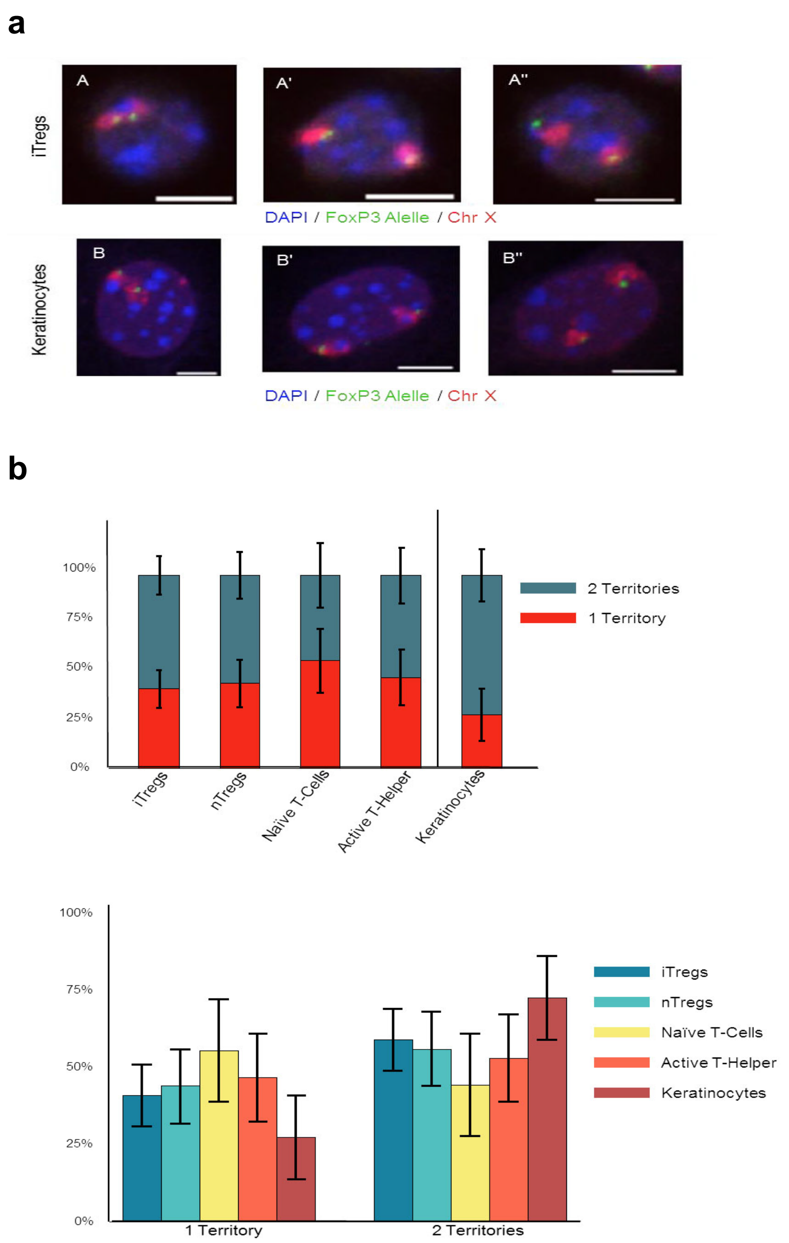

Supplemental Figure 1. (a) 3D maximum intensity projections of mouse induced Tregs (upper panel) and keratinocyte (lower panel) nuclei (blue) with FOXP3 (green) and chromosome X (red) labeled during X separation and coalescence. **(b)** 3D analysis of chromosome X coalescence frequency in various mouse cell types. Values presented as mean error bars are 95% confidence intervals of the mean, N> 41 nuclei. Values are a single biological replicate.

Supplemental Figure 2

a

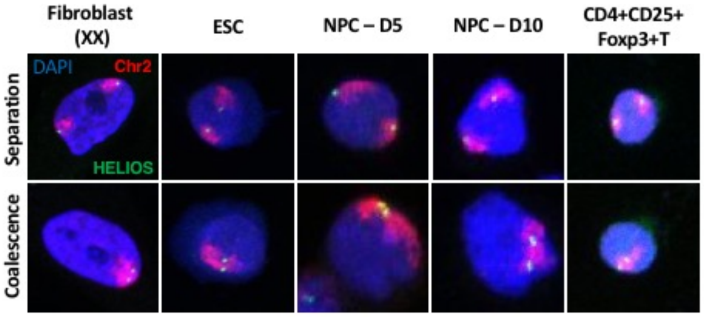

b

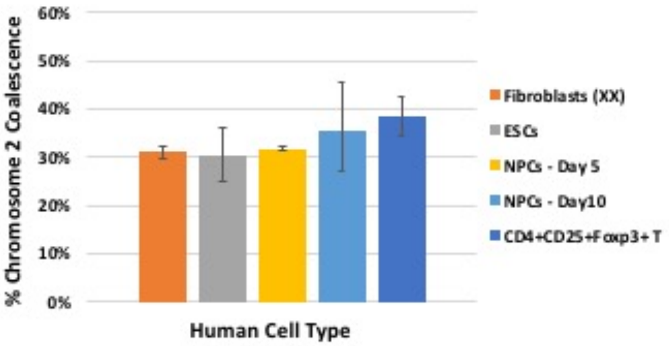

Supplemental Figure 2. (a) 3D DNA FISH maximum intensity projections of human nuclei labeled with DAPI (blue), chromosome 2 (red), and chromosome 2 gene loci, HELIOS (green) in various human cell types containing two chromosome 2, during separation (upper panel) or coalescence (lower panel). **(b)** 3D analysis of chromosome 2 coalescence frequency in various human female cell types. Values presented as mean ± standard deviation. **P*<0.05, ***P*<0.01, Student's *t*-test. Each column represents approximately 150-200 nuclei analyzed.

Supplemental Figure 3

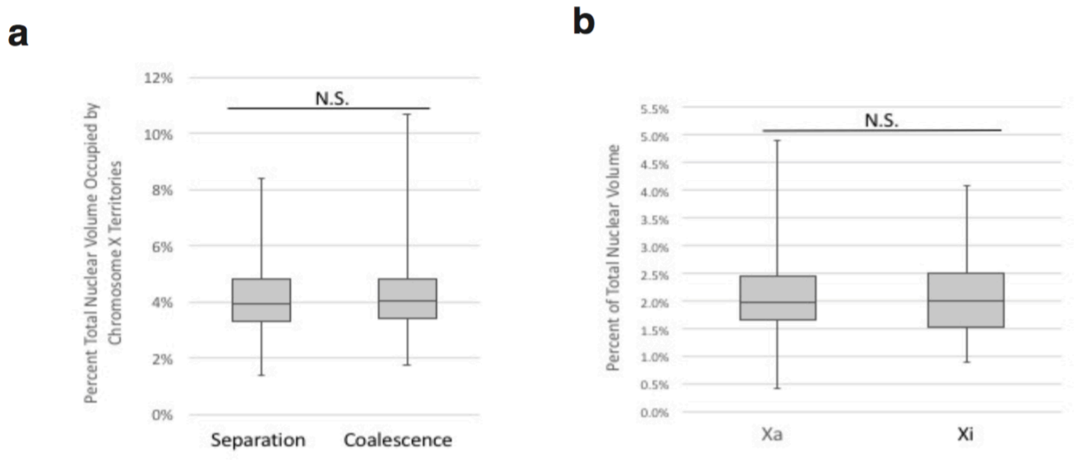

Supplemental Figure 3. (a) Percent total IMR- nuclear volume occupied by X chromosomes during either separation or coalescence. *P<0.05, **P<0.01, Student's t-test. Each whisker-box plot represents 100 nuclei analyzed. **(b)** Percent total IMR-90 nuclear volume occupied by either the Xi or Xa during chromosome X separation. *P<0.05, **P<0.01, ***P<0.001, Student's t-test. A total of 100 nuclei were analyzed. N.S. = Not Significant.
